# A comparative investigation on H3K27ac enhancer activities in the brain and liver tissues between wild boars and domesticated pigs

**DOI:** 10.1111/eva.13461

**Published:** 2022-08-16

**Authors:** Zhimin Zhou, Tao Jiang, Yaling Zhu, Ziqi Ling, Bin Yang, Lusheng Huang

**Affiliations:** ^1^ State Key Laboratory of Swine Genetic Improvement and Production Technology Jiangxi Agricultural University Nanchang China

**Keywords:** ChIP‐Seq, domestication, H3K27ac, mRNA, pig

## Abstract

Dramatic phenotypic differences between domestic pigs and wild boars (*Sus scrofa*) provide opportunities to investigate molecular mechanisms underlying the formation of complex traits, including morphology, physiology and behaviour*.* Most studies comparing domestic pigs and wild boars have focused on variations in DNA sequences and mRNA expression, but not on epigenetic changes. Here, we present a genome‐wide comparative study on H3K27ac enhancer activities and the corresponding mRNA profiling in the brain and liver tissues of adult Bama Xiang pigs (BMXs) and Chinese wild boars (CWBs). We identified a total of 1,29,487 potential regulatory elements, among which 11,241 H3K27ac peaks showed differential activity between CWBs and BMXs in at least one tissue. These peaks were overrepresented by binding motifs of *FOXA1*, *JunB*, *ATF3* and *BATF*, and overlapped with differentially expressed genes that are involved in female mating behaviour, response to growth factors and hormones, and lipid metabolism. We also identified 4118 nonredundant super‐enhancers from ChIP‐Seq data on H3K27ac. Notably, we identified differentially active peaks located close to or within candidate genes, including *TBX19, MSTN, AHR* and *P2RY1,* which were identified in DNA sequence‐based population differentiation studies. This study generates a valuable dataset on H3K27ac profiles of the brain and liver from domestic pigs and wild boars, which helps gain insights into the changes in enhancer activities from wild boars to domestic pigs.

## 1 INTRODUCTION

Domestication of farm animals is a milestone of human society, and has resulted in dramatic phenotypic changes in body length, coat colour, reproduction and aggressive behaviour in domestic pigs (Amaral et al., 2011; Chao et al., 2015; Rubin et al., 2012; Wang et al., 2015; Zhu et al., 2017). Population differentiation analysis between wild boars and domestic pigs has revealed a number of loci close to candidate genes that are potentially associated with phenotypic variation in pigs, including *NR6A1* and *LOCRL* associated with the body length in European domestic pigs (Rubin et al., 2012); *MC1R* and *KIT* associated with the coat colour in Mediterranean pigs (Fontanesi & Russo, 2013); *PRM1*, *PRM2* and *JMJD1C* genes related to the reproductive traits in Chinese pig breeds (Wang et al., 2015); and *TBX19* related to the timidity trait in Chinese domestic pigs (Zhu et al., 2017). Most of the differentiated loci are located in intron and intergenic regions, suggesting their potential role in regulating gene expression.

Recently, disease‐associated noncoding single nucleotide polymorphisms (SNPs) have been shown to be significantly enriched in enhancer elements and explain a larger proportion of phenotypic variation than do the coding variants (Gusev et al., 2014). These enhancer variants may influence transcriptional output, thereby offering a mechanistic basis for explaining their association with complex traits and common diseases (Corradin & Scacheri, 2014). For instance, a noncoding SNP (rs7539120) was identified, which maps within an enhancer of *NOS1AP* and affects cardiac function by increasing *NOS1AP* expression (Kapoor et al., 2014). A regulatory region of *KITLG* was dissected, which contains an enhancer SNP, contributing to the classic blond hair phenotype in northern Europeans (Guenther et al., 2014). Enhancers that overlap with obesity‐associated *FTO* intron variants regulate *IRX3* expression to influence body mass and composition phenotypes (Smemo et al., 2014). Alternatively, investigation on the evolution of active liver enhancers across 20 mammalian species demonstrated their central roles in evolutionary processes across mammals (Villar et al., 2015).

As the central nervous system of memory, cognition and awareness, the brain is responsible for critical phenotypic changes, such as tamed behaviour during domestication and artificial selection (Carneiro et al., 2014; Hosoya et al., 2018). The liver is the major organ for detoxifying various metabolites, synthesizing proteins and producing biochemicals necessary for digestion, and plays a core role in growth of pigs (Elias & Bengelsdorf, 1952). Therefore, the liver could also be subject to artificial selection in response to dramatic changes in diet of wild boars and domesticated pigs.

**FIGURE 1:**
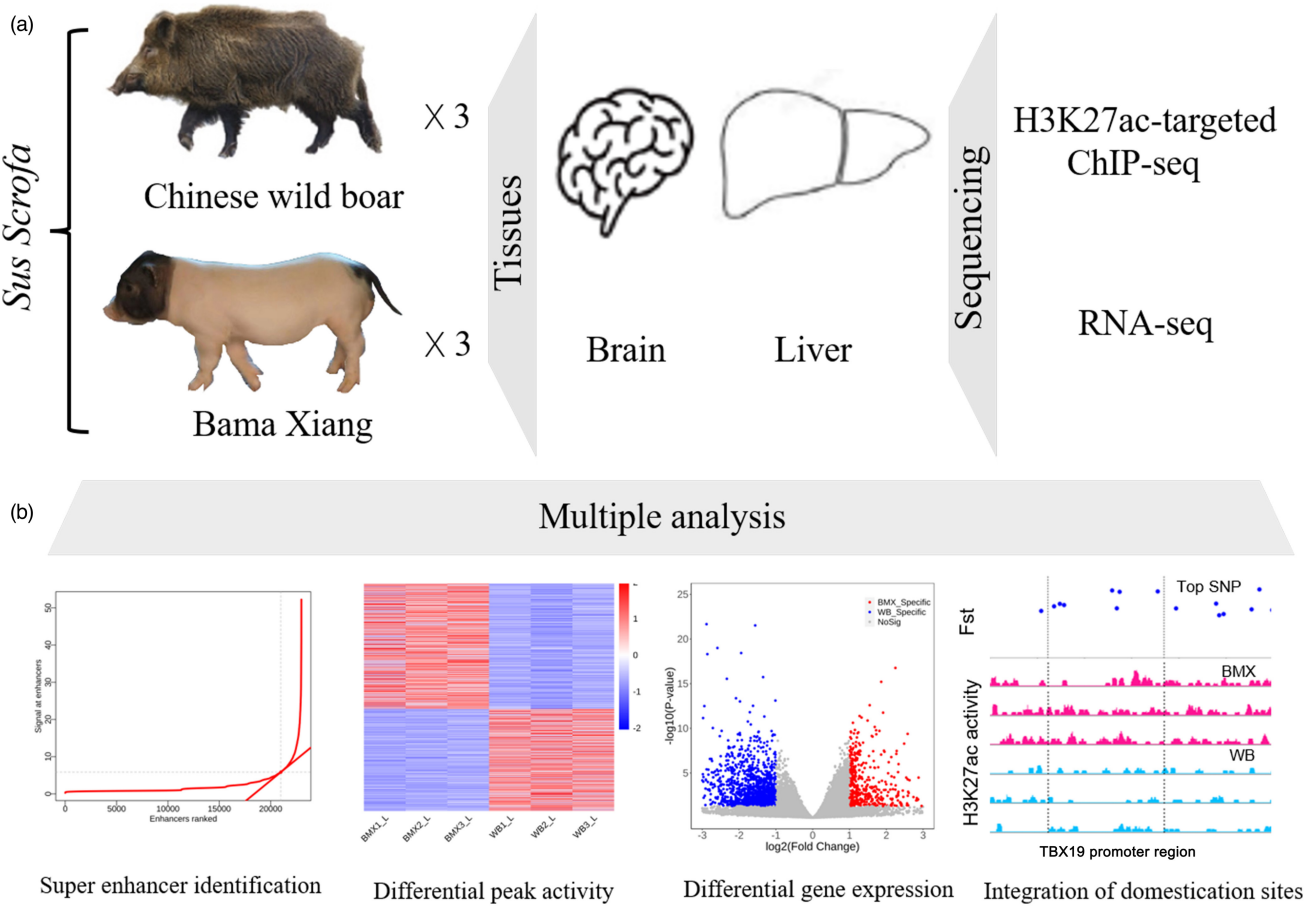
Overall experimental design and analysis pipeline. (a) Individual pigs (Bama Xiang and Chinese wild boar) and their samples (the brain and liver) investigated in this study. (b) Four modules of analyses were performed in this study: the identification of super‐enhancers, differential peak activity analysis, differential gene expression analysis and integrative analysis of domestication loci.

Currently, very limited studies have investigated the differences in regulatory element activity between domesticated pigs and wild boars. In this study, we applied H3K27ac‐targeted chromatin immunoprecipitation with high‐throughput sequencing (ChIP‐Seq) to identify the differential H3K27ac regions and super‐enhancers in the brain and liver tissues of Bama Xiang pigs (BMXs) and Chinese wild boars (CWBs), and integrated the differentially expressed genes derived from RNA sequencing to characterize the potential regulatory mechanisms in the brain (cerebral cortex) and liver (left outside hepatic lobe) that are associated with domestication or artificial selection (Figure 1a,b).

## 2 MATERIALS AND METHODS

### 2.1 Experimental animals and tissue collection

Tissues from the brain and liver of three biological replicates of each of adult CWBs and BMXs were carefully dissected following standardized sample collection protocols of the FAANG Project (https://www.faang.org).

### 2.2 Chromatin immunoprecipitation sequencing

SimpleChIP® Plus Enzymatic Chromatin IP Kit (Magnetic Beads, 9005) was used to perform chromatin immunoprecipitation with 500 μg of chromatin and 5 μg of H3 lysine 27 acetylation antibody (H3K27ac) (active motif, 39133) according to the protocols from https://www.encodeproject.org/about/experiment‐guidelines/and https://www.animalgenome.org/community/FAANG. Briefly, 37% formaldehyde was used to treat the dissected tissues for covalent cross‐linking of proteins with DNA. This was followed by cell disruption and sonication to shear the chromatin to a target size of 100–300 bp (Landt et al., 2012; Shen et al., 2012). Then the modified histones with bound DNA were enriched using the corresponding antibody. After protein removal, the DNA was purified, and real‐time quantitative polymerase chain reaction was performed. ChIP and input (control sample) library construction and sequencing of ChIP and input samples (control) were carried out according to Illumina protocols with minor modifications (Illumina).

Clean reads were mapped to the pig reference genome Sscrofa 11.1 (http://hgdownload.cse.ucsc.edu/goldenPath/susScr11/bigZips/) using the Burrows‐Wheeler Aligner (Abuín et al., 2015), allowing two mismatches. The duplicated reads were removed using SAMtools. Then, Model‐based Analysis for ChIP‐Seq (MACS version 2.1.0) peak caller was applied to infer the histone modification regions (Zhang et al., 2008), that is H3K27ac peaks, with the following parameters: *‐t Sample_ac_R1_sorted.bam ‐c Sample_input_R1_sorted.bam ‐‐broad ‐g 2.48e9 ‐‐broad‐cutoff 0.1 ‐n Sample_ac*. The peaks identified in individual samples were combined by intersecting all peaks across data sets using the bedtools version 2.27.0 (Quinlan & Hall, 2010). The bamCorrelate module of deepTools (Ramírez et al., 2016) was used to calculate the Spearman correlation coefficients of the BAM files from ChIP‐Seq data of every sample. In addition, library complexity statistics (Non‐Redundant Fraction [NRF], PCR Bottlenecking Coefficient 1 [PBC1] and PBC2) and the fraction of reads in peaks (FRiP) values were calculated using the bedtools bamtobed and multicov and SAMtools flagstat modules.

### 2.3 RNA‐Seq

Total RNA was isolated from tissues using TRIzol reagent (Invitrogen) and used to construct cDNA libraries using a NEBNext® UltraTM Directional RNA Library Prep Kit for Illumina (New England Biolabs). Approximately, 47.1 million paired‐end reads (150 PE) on an average were generated using the Illumina HiSeq 4000 platform.

Raw reads with more than 10% N base contents were removed. Clean reads were mapped to the pig reference genome *Sscrofa* 11.1 (http://hgdownload.cse.ucsc.edu/goldenPath/susScr11/bigZips/) using STAR‐2.5.3a (Dobin et al., 2013). An average of 47.1 million reads were generated for RNA‐Seq samples, except for a liver sample of the wild boar 3 (Table S1), 11 out of 12 samples that assayed with H3K27ac ChIP‐Seq were also profiled with RNA‐Seq, among which 86.6% data were uniquely mapped to the reference genome on average. The transcripts were assembled and merged with Stringtie with reference to Ensembl Gene transfer format (GTF) (98.111), which was further merged with Ensembl GTF (98.111) to obtain a customized GTF file as the reference to quantify gene expression. Gene levels were quantified using FeatureCounts (Liao et al., 2014). After removing the mitochondrial genes, the Reads Per Kilobase Million (RPKM) algorithm was used to normalize the expression of each sample based on the gene length and read count mapped to the gene. Differential expression was analysed using the DESeq2 R package (Love et al., 2014). Genes with *p* value ≤0.05 and fold change ≥2 were assigned as differentially expressed genes.

### 2.4 Annotations of putative promoters and enhancers

Different genomic features and transcription factor‐binding sites (TFBS) were enriched using the HOMER program (http://homer.ucsd.edu/homer/motif/motifDatabase.html) (Heinz et al., 2010). Transcription start site (TSS) enrichment was analysed using deepTools bamCoverge module. ClueGO (Bindea et al., 2009) was used to analyse gene ontology (GO) enrichment. The *p* values for enrichment of GO terms were corrected using the Benjamini–Hochberg approach.

### 2.5 Identification of peaks with differential activities between BMXs and CWBs

The H3K27ac peaks were merged using the merge command in bedtools (Quinlan & Hall, 2010). Read depths in a peak region were calculated using the SAMtools bedcov utility (version 1.2) and normalized with respect to the RPKM value using the DESeq2 R package (Love et al., 2014); the normalized value was defined as the peak activity. We used the DESeq2 program to identify peaks that showed differential activities between the two breeds. Regions with *p* value ≤0.05 and fold change ≥2 were assigned as peaks with differential activity.

### 2.6 Identification of super‐enhancers

Super‐enhancers were identified using the ROSE algorithm (Whyte et al., 2013) in all H3K27ac samples. The definition and normalization procedures were the same as that used for all peaks of H3K27ac.

### 2.7 Detection of SNPs and STRs of population differentiation

We downloaded the results of Fst/Rst analysis on 19,249,347 SNPs and 296,831 STRs (Short Tandem Repeats) from 157 domestic pigs and 24 wild boars (Table S5) from Wu et al. (2021). The fixation index (Rst) (Slatkin, 1995) of STRs was defined as: Rst = (St − Sw)/St, where Sw is the average sum of squares of the differences in allele length within each population, and St is the average sum of the squares of the differences in allele length across all individuals under investigation. Rst values were Z‐transformed. The top 1% SNP/STRs with the highest Fst/Rst values were checked for their intersection with H3K27ac signals.

## 3 RESULTS

### 3.1 Genome‐wide mapping of H3K27ac regions in BMXs and CWBs

Here, we performed H3K27ac‐targeted ChIP‐Seq of liver and brain tissues from BMXs, a typical indigenous pig breed from Southern China pigs (132–141 days, *n* = 3) and CWBs, from Southern China (40–60 kg, *n* = 3). The average number reads uniquely mapped to the reference genome was 27.8 M, ranging from 19.6 to 36.7 M. The average NRF, PBC1, PBC2 and FRiP values of the samples were 0.934, 0.938, 20.074 and 0.374, respectively, surpassing the data quality standards from the ENCODE project (https://www.encodeproject.org/), which validated the ChIP‐Seq data (Table S1). We identified an average of 56,281 peaks from 12 H3K27ac ChIP‐Seq samples and merged them into a union of 129,487 peaks. Clustering analysis based on the activity of the 129,487 peaks revealed that the samples were first grouped by two tissues, and then by CWB and BMX breeds (Figure S1A), which indicates that H3K27ac activity profile of the liver or brain was sufficient to separate BMXs from CWBs. The 129,487 H3K27ac peaks were further grouped into 13,207 proximal peaks intersected with a ± 1 kb region from the transcription start site of any transcript provided by the Ensembl database (release 98) and 116,280 distal peaks. Next, we examined the enrichment of genomic features with the proximal and distal peaks. The proximal peaks of H3K27ac were mostly located in introns (56.89%) and promoter (22.94%) regions, whereas the distal peaks were in introns (63.41%) and intergenic regions (28.41%) (Figure S1B,C). Meanwhile, H3K27ac signals were enriched in gene transcription start sites (Figure S1D).

### 3.2 Differential H3K27ac peak activity between BMXs and CWBs

**FIGURE 2:**
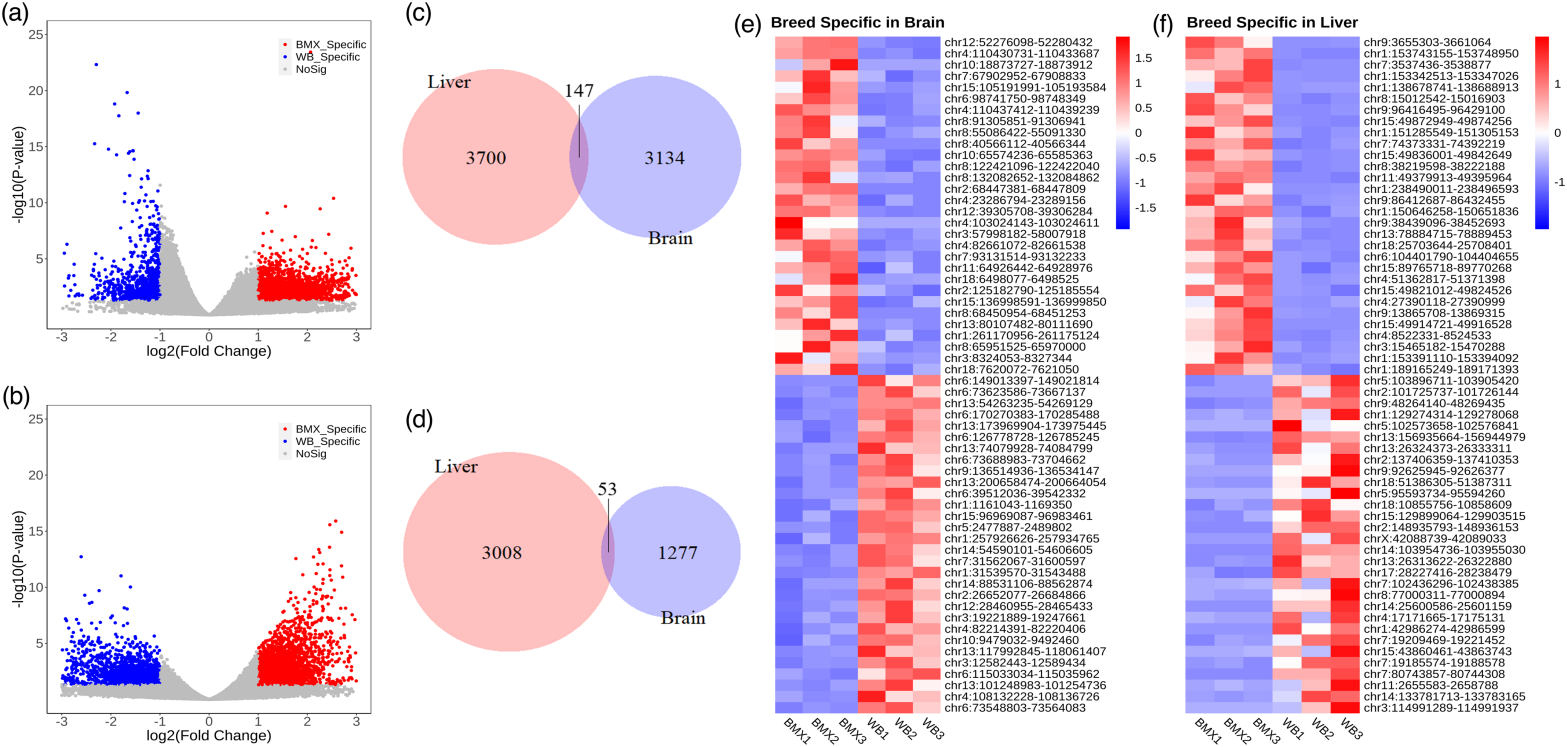
Analysis of peaks with differential activities. (a,b) Volcano plot showing the differential activity analysis of peaks in brains (a) and livers (b) of BMXs and CWBs. (c,d) The intersection of (c) BMX‐ and (d) CWB‐specific peaks in the brain and liver. (e,f) Unsupervised hierarchical clustering of the top differential peaks in (e) the brain and (f) the liver of BMXs and CWBs.

We next identified H3K27ac peaks that showed differential activities in brain and liver tissues between BMXs and CWBs. At the threshold of fold change ≥2 and *p* ≤ 0.05, we identified 6981 high activity peaks (3281 in the brain, 3847 in the liver, 147 in both tissues) in BMXs and 4338 high activity peaks (1330 in the brain, 3061 in the liver and 53 in both tissues) in CWBs (Figure 2a–d). The limited overlap of the differential active peaks in the two tissues reflects tissue‐specific changes in enhancer activity between wild boars and domestic pigs. The top 60 peaks that showed the most significant differential activities between BMXs and CWBs in the two tissues are presented in Figure 2e,f. At the same threshold, we identified 3035 genes that showed comparatively high expression in BMXs (447 in the brain, 2680 in the liver and 92 in both tissues) and 3045 genes that showed comparatively high expression in CWBs (1345 in the brain, 1997 in the liver and 297 in both tissues) (Figure S2). Among these genes, we identified 2142 highly expressed genes in BMXs (336 in the brain and 1806 in the liver) and 1809 highly expressed genes in CWBs (533 in the brain and 1276 in the liver), which had at least one differentially active peak located within 500 kb of the transcription start sites. The highly expressed genes in the brain of BMXs were enriched in pathways related to type I interferon response (*p*‐value = 3.61 × e^−4^), female mating behaviour (*p*‐value = 3.66 × e^−3^) and reproductive behaviour (*p*‐value = 0.03). The highly expressed genes in the liver of BMXs were enriched in pathways related to lipid metabolism (*p*‐value = 1.70 × e^−13^), liver development and regeneration (*p*‐value = 2.82 × e^−3^), and hormone metabolism (*p*‐value = 0.04). The highly expressed genes in the brain of CWBs were enriched in pathways associated with cerebellar cortex development (*p*‐value = 3.49 × e^−4^), response to growth factors (*p*‐value = 1.82 × e^−3^), and T‐helper 1‐type immune response (*p*‐value = 0.05). The highly expressed genes in the liver of CWBs were enriched in pathways related to response to hormones (*p*‐value = 3.78 × e^−5^), pigment biosynthesis (*p*‐value = 0.02) and growth regulation (*p*‐value = 0.02) (Figure S3; Tables S2 and S3).

Transcription factors (TFs) play essential roles in triggering epigenetic reprogramming (Sindhu et al., 2012). Therefore, we investigated the enrichment of TF‐binding motifs in peaks that showed differential H3K27ac activity between BMXs and CWBs using the HOMER program (http://homer.ucsd.edu/homer/motif/motifDatabase.html) (Heinz et al., 2010). We found 11 (FOXA2, FOXA1, IRF3, GATA4, IRF4 and GATA6 for high activity peaks of BMXs, and BATF, ATF3, JunB, ERG and FOXO1 for high activity peaks of CWBs) significantly enriched TF‐binding motifs (q ≤ 0.05) (Table S4). FOXA1 is located in different genetic regions between domesticated pigs and wild boars (Yang, Cui, et al., 2017; Yang, Yan, et al., 2017), and FOXA1 and JunB interact with *ESR1* (Bernardo et al., 2010; Teyssier et al., 2001), a gene that was involved in pig reproduction (Saraswat et al., 2020).

### 3.3 Identification of super‐enhancers in the brain and liver of BMXs and CWBs

**FIGURE 3:**
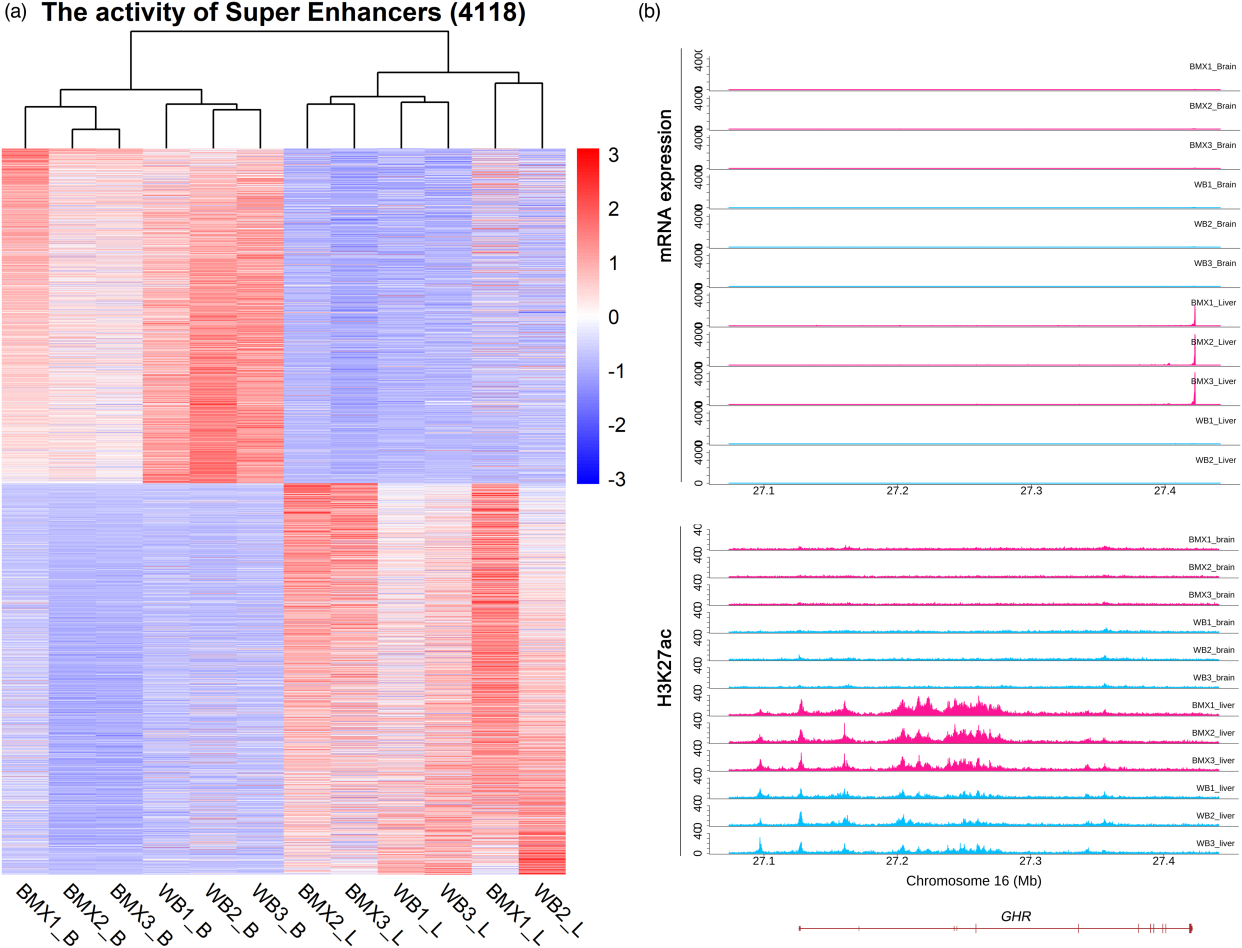
Diversity of super‐enhancers. (a) Unsupervised hierarchical clustering of 4118 super‐enhancers. (b) The tracks of H3K27ac activity and genes in the representative super‐enhancer that show differential activities between BMXs and CWBs for reference (H3K27ac: chr16:27093775–27396751 Gene: *GHR*).

Multiple adjacent enhancer elements can be clustered into super‐enhancers, which are the major drivers of transcriptional activation (Lovén et al., 2013). We identified 1682 super‐enhancers on an average in each sample (Figure S4). After merging, we obtained 4118 nonredundant super‐enhancers. We next investigated the differences in super‐enhancer activities between BMXs and CWBs in the two tissues and identified 64 super‐enhancers with differential activities (Figure 3a), including 50 high‐activity super‐enhancers (3 in the brain and 47 in the liver) in BMXs and 14 high‐activity super‐enhancers (2 in the brain and 12 in the liver) in CWBs. Among different super‐enhancers, a high activity super‐enhancer (chr16:27093775–27396751) located in *GHR* in the brain of BMXs was associated with a high expression of *GHR* (Figure 3b), a gene involved in the positive regulation of organism development (Godowski et al., 1989), and defects in this study are involved in growth regulation (Wu et al., 2020). In addition, a high‐activity super‐enhancer (chr14:88513242–88574880) in the liver of BMXs overlapped with *GDF10* (Figure S5), a gene associated with embryonic development (Zhao et al., 1999).

### 3.4 Integration analysis of domestication loci with differential H3K27ac regions

**FIGURE 4:**
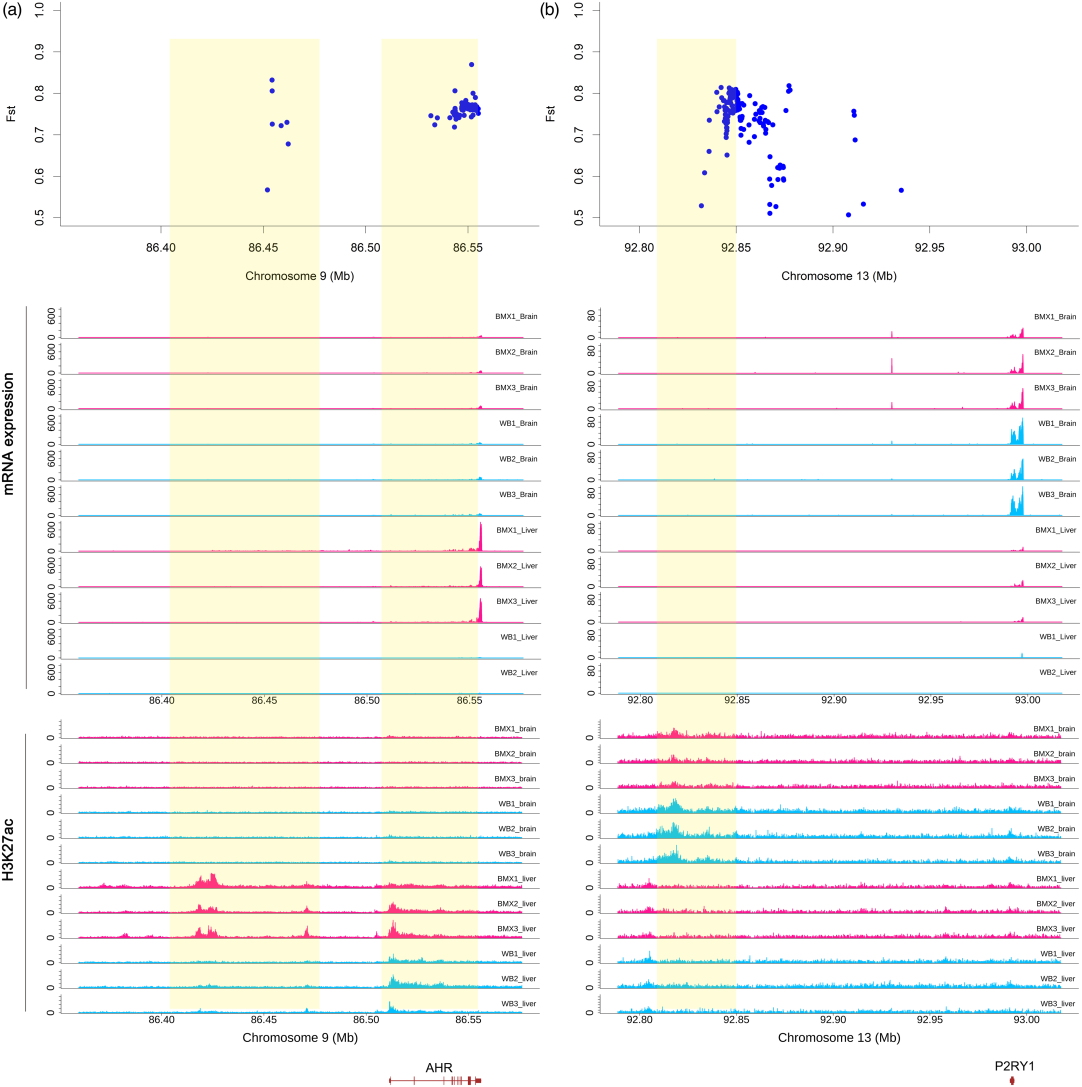
Overlap of differentially active H3K27ac peaks with the previously reported differentiated loci and candidate genes. (a) In BMXs, six high‐activity peaks (chr9:86379472–86384542, chr9:86412687–86432455, chr9:86450583–86455826, chr9:86458600–86459756, chr9:86462254–86478890 and chr9:86504316–86506928) were upstream of *AHR*. (b) In CWBs, two high activity peaks (chr13:92808713–92821750 and chr13:92849261–92850154) were upstream of *P2RY1*. The yellow shades mark differential H3K27ac regions between BMXs and CWBs.

To further explore the role of regulatory elements in pig domestication, we compiled 78 genes that were reported to be highly associated with domestication (Wu et al., 2021; Yang, Cui, et al., 2017; Yang, Yan, et al., 2017; Zhu et al., 2017) and the top 1% highly differentiated variants (SNPs and STRs) between wild boars and domestic pigs (Wu et al., 2021) (Figure S6). We examined their overlaps with the differentially active H3K27ac peaks of BMXs and CWBs identified in this study (Tables S6–S8). Interestingly, 251 differential H3K27ac peaks between BMXs and CWBs were located within 500 kb of the transcription start sites of 63 genes (Table S9); representative examples are listed in Table 1. For example, we identified six high activity peaks (chr9:86379472–86384542, chr9:86412687–86432455, chr9:86450583–86455826, chr9:86458600–86459756, chr9:86462254–86478890 and chr9:86504316–86506928) upstream of *AHR* in the liver of BMXs (Figure 4a), which displayed higher expression in the liver of BMXs than that of CWBs. *AHR* plays a critical role in pig reproduction and ovary development by affecting the hypothalamus–pituitary–gonadal axis (Baba et al., 2005). Moreover, in the liver of BMXs, we identified an overlap of a high peak (chr15:94395185–94404081) with a significantly differentiated STR (chr15:94401981–94401998) and several SNPs located close to *MSTN* (Figure S7A and S8; Table S11) that plays a key role in muscle growth (Aiello et al., 2018). In the brain of BMXs, three high‐activity peaks (chr4:82658612–82659843, chr4:82661072–82661538 and chr4:82664040–82664615) were located near *TBX19* (Figure S7B), a gene located in the top differentiated regions between Chinese domesticated pigs and wild boars (Zhu et al., 2017). In the brain of CWBs, two high‐activity peaks (chr13:92808713–92821750 and chr13:92849261–92850154) were upstream of *P2RY1* (Figure 4b), which displayed higher expression in the CWB brain than that in the BMX brain; this gene is associated with the nervous and sensory systems (Okaty et al., 2020; Prescott et al., 2020). Moreover, we identified differential H3K27ac peaks (chr1:13930748–13935472, chr8:55086422–55091330, chr14:47922638–47934516 and chr18:20373534–20376561) within 500 kb of the transcription start sites of *ESR1, NMU*, *PATZ1* and *LEP* (Figure S9A–D) that were previously proposed to be domestication‐associated genes (Graham et al., 2003; Luo et al., 2015; Mankowska et al., 2015; Yang et al., 2010). Other notable overlaps of the differentiated SNPs with the differentially active peaks are shown in Table S10.

**TABLE 1:** Representative differential H3K27ac peaks adjacent to domestication‐related genes

Chr	Start	End	Log_2_Fold_Change (BMX/CWB)	*p*‐Value	Gene	Function
chr15	94395185	94404081	1.136	2.64E‐03	MSTN	Muscle growth
chr4	82658612	82659843	1.394	1.63E‐03	TBX19	Timidity behaviour; Developmental process
chr4	82661072	82661538	2.081	9.04E‐07	TBX19	
chr4	82664040	82664615	1.679	4.84E‐03	TBX19	
chr13	92808713	92821750	−1.016	2.73E‐09	P2RY1	Nervous system
chr13	92849261	92850154	−1.587	3.70E‐05	P2RY1	
chr9	86379472	86384542	1.946	1.54E‐08	AHR	Reproduction traits
chr9	86412687	86432455	2.215	9.00E‐13	AHR	
chr9	86462254	86478890	1.177	2.35E‐04	AHR	
chr9	86450583	86455826	1.500	1.07E‐04	AHR	
chr9	86458600	86459756	1.273	1.73E‐02	AHR	
chr9	86504316	86506928	1.158	8.79E‐03	AHR	
chr1	13930748	13935472	1.228	9.48E‐04	ESR1	Puberty and reproduction
chr14	47922638	47934516	−1.187	7.91E‐09	PATZ1	
chr8	55086422	55091330	1.277	3.56E‐08	NMU	Energy balance
chr18	20373534	20376561	−1.017	6.29E‐03	LEP	

## 4 DISCUSSION

In this study, we generated activity landscapes of H3K27ac from two important tissues (the brain and liver) from BMXs and CWBs. In total, we identified 129,487 candidate regulatory elements in the pig genome, including 13,207 potential promoters, 116,280 putative enhancers and 4118 super‐enhancers. The observation that the H3K27ac peaks were mainly enriched in introns, which underscores the important role of intronic regions close to the 5′ end of genes in the regulation of gene transcription, is in agreement with reports in other species, such as rats (Chan et al., 1999) and Arabidopsis (Gallegos & Rose, 2017). To the best of our knowledge, this is the first study reporting H3K27ac signals in the brain and liver of Chinese wild boars, the samples of which are relatively hard to access. Among the high‐activity peaks identified in the brain and liver of CWBs, only 19.40% and 42.21% overlapped with the peaks previously identified from brain and liver tissue (Zhao et al., 2021), and only 25.79% and 26.85% overlapped with those in another study, respectively (Zhu et al., 2022), therefore, highlighting the dataset as a novel resource that contributes to the argument of pig H3K27ac activity repositories.

The observation that the samples were clustered first by tissues and the majority of differentially active peaks were tissue‐specific was in line with previous findings showing that the tissues are major sources of variations in peak activities (Nord et al., 2013; Zhu et al., 2022). By integrating ChIP‐Seq and RNA‐Seq data, we identified several differentially expressed genes and active peaks in the liver and brain between BMXs and CWBs, indicating extensive changes in enhancer activities and gene expression in these tissues during domestication. These genes were enriched in reproductive behaviour, growth factors, fatty acid metabolism and immune‐related pathways, reflecting phenotypic and physiological differences between BMXs and CWBs, such as increased fecundity and growth rate during domestication (Rubin et al., 2012; Wang et al., 2011).

In addition, we obtained 11 transcription factors that were significantly enriched in the differential H3K27ac region between BMXs and CWBs, among which, FOXA1 and JunB were reported to interacted with ESR1 (Bernardo et al., 2010; Teyssier et al., 2001), a gene that encodes oestrogen receptors and was related to the reproductive traits of pigs (Saraswat et al., 2020).

Notably, among the differentially active super‐enhancers, we identified a super‐enhancer in *GHR* in the liver, which showed greater activity coinciding with higher expression in BMXs than in wild boars. The liver‐specific activity of the super‐enhancer is consistent with liver‐biased expression of *GHR*. Notably, *GHR* is an important growth regulator in different species (Yang, Cui, et al., 2017; Yang, Yan, et al., 2017). Our results suggest that the super‐enhancers could play a role in enhancing the growth of BMXs by increasing *GHR* expression.

We observed several cases in which the differential activity peaks were located close to candidate genes (*AHR, MSTN, TBX19* and *P2RY1*) that are close to the differentiated loci between domestic pigs and wild boars. In the liver of BMXs, we identified several high H3K27ac activity peaks that cover many highly differentiated SNPs (Wu et al., 2021) located upstream of *AHR* that plays a critical role in reproduction (Baba et al., 2005). Interestingly, the haplotypes in *AHR* region in European commercial pigs introgressed from Asian pigs, were subjected to artificial selection, and were associated with an increased litter size (Bosse et al., 2014). The increased enhancer activity could upregulate *AHR* expression, which in turn contributes to improved fertility traits in Chinese domestic pigs compared with that in wild boars. In BMXs, we identified high‐activity peaks overlapped with highly differentiated SNPs located within the promoter region of *TBX19* that was repeatedly shown to be the highest differentiated locus between Chinese domestic pigs and wild boars (Zhu et al., 2017). We hypothesized that genomic variation affects the *TBX19* promoter activity in BMXs. Notably, we did not observe a significantly differential expression of *TBX19* in the brain (cerebral cortex) between BMXs and CWBs. *TBX19* was reported to be exclusively expressed in the pituitary gland and was required for the expression of *POMC* in pituitary corticotrophs (Liu et al., 2001); we thereby hypothesize that *TBX19* in the pituitary gland could be differentially expressed between domestic pigs and wild boars. We identified higher *P2RY1* expression and nearby enhancer activities in the brain of CWBs than that of BMXs along with an important role of *P2RY1* in regulating eating behaviour (Kittner et al., 2006; Yu et al., 2021), which may explain the differences in eating behaviour between wild boars and demonstrated pigs. Nevertheless, these observations provide promising clues and require further experimental validation.

In conclusion, the generated H3K27ac profiles and RNA‐Seq data in the brain and liver of BMXs and CWBs provide valuable resources for exploring the dynamic changes in gene transcription from wild boar to domestic pigs during or after domestication. The identified H3K27ac peaks and super‐enhancers are useful for interpreting the genetic loci under selection or associated with complex traits in pigs and provide valuable resources for cross‐species comparative analysis.

## FUNDING INFORMATION

This work was supported by was supported by the National Natural Science Foundation of China (32160781).

## CONFLICT OF INTEREST

The authors declare no competing interests.

## Supporting information


Figures S1–S9

Tables S1–S11

## Data Availability

All RNA‐Seq and ChIP‐Seq data in this study are available at CNCB (http://www.cncb.ac.cn) with accession number: CRA007675.
