# Supplementary material for: A comparative investigation on H3K27ac enhancer activities in the brain and liver tissues between wild boars and domesticated pigs
Source: Evol Appl. 2022 Aug 16;15(8):1281–90. doi: 10.1111/eva.13461 (PMC9423090; doi:10.1111/eva.13461)
Supplement: Supplementary file 1 — Figures S1–S9 [file EVA-15-1281-s001.docx]

**Supplementary Figures**

**A comparative investigation on H3K27ac enhancer activities in the brain and liver tissues between wild boars and domesticated pigs**

The PDF file includes:

Supplementary figures: Fig. S1 – S9


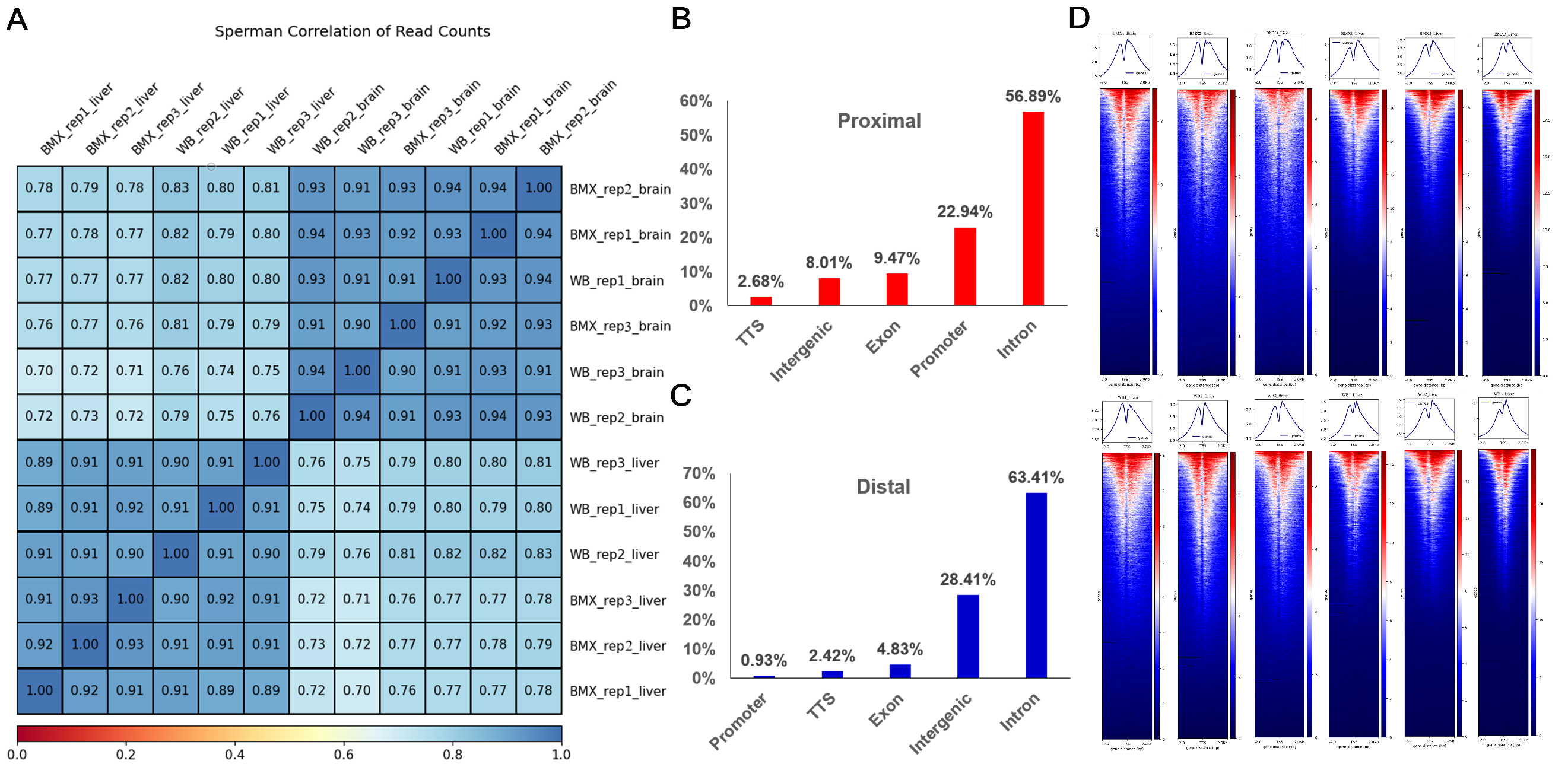


Figure S1. The identification and annotation of H3K27ac regions in brain and liver of Bama Xiang pigs (BMXs) and Chinese wild boars (CWBs). (A) The heatmap of lineage-specific histone modification in brain and liver across BMX and CWB. (B-C) Distribution of H3K27ac peaks across genome. (D) Heatmaps depicting normalized ChIP-seq signal (H3K27ac) at 2 kb near the TSS, sorted by signal intensity.


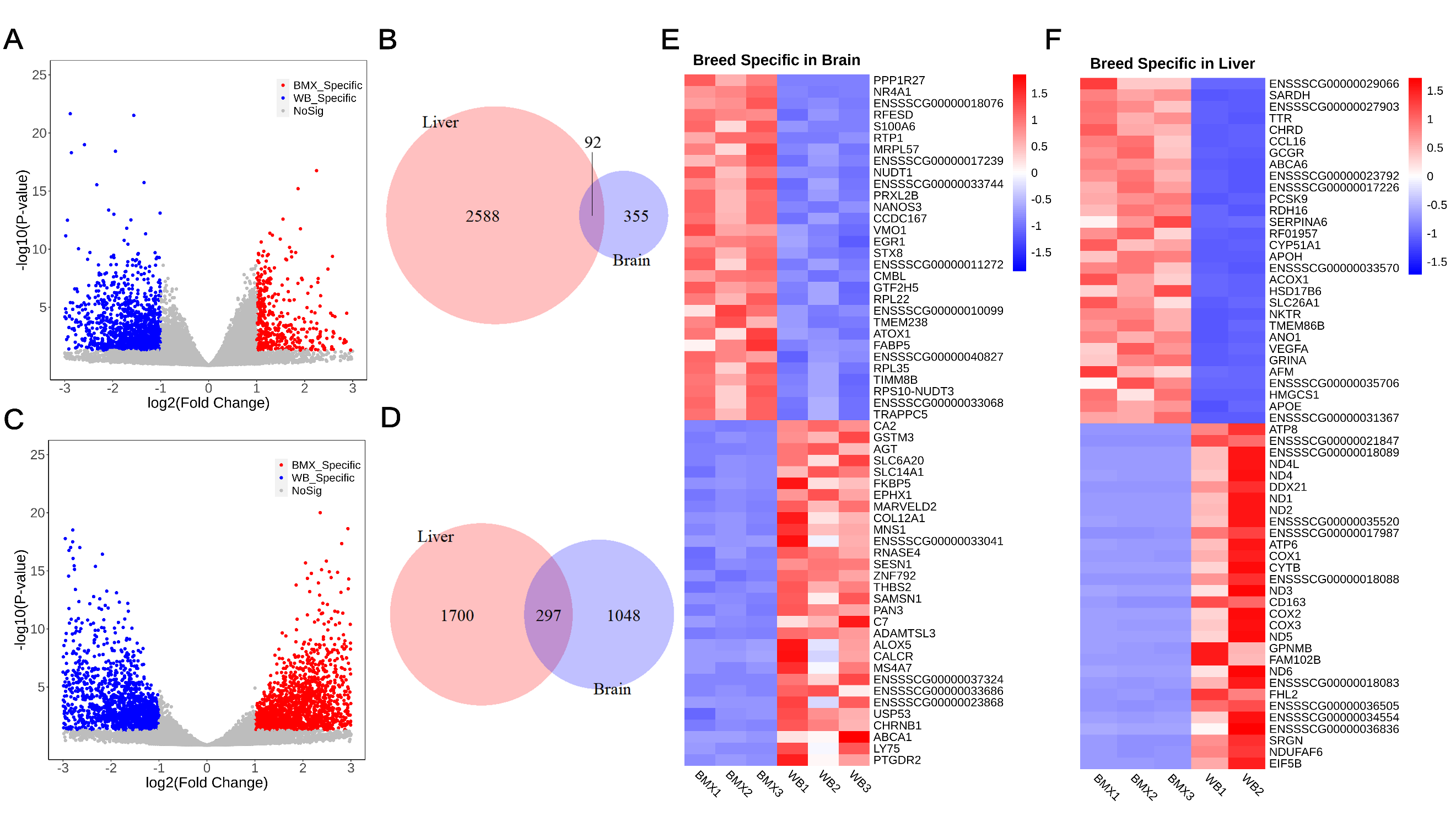


Figure S2. Analysis of differentially expressed genes. (A-B) Volcano plot showing the differential expression analysis of genes between BMX and CWB from brain (A) and liver (B). (C-D) The intersection of BMX (C), CWB (D) specific genes in brain and liver. (E-F) Unsupervised hierarchical clustering of the top differential genes between BMX and CWB in brain (E) and liver (F). Among lead differentially expressed genes that shown higher expression in BMX pigs, EGR1 and RTP1 was related to brain development and neuronal activity (Sun et al., 2019; Zhongdong et al., 2017); FABP5 and ACOX1 have been linked to lipid metabolism (Griffin et al., 2020; Senga et al., 2018); CYP51A1 was involved in birth weight and maternal lipid levels (Lewinska et al., 2013). For CWB high expressing genes, ABCA1 was associated with brain cholesterol uptake and neuronal structure and function (Karasinska et al., 2009); ADAMTSL3 was related to height and body mass index (Liu et al., 2010); PTGDR2 and DDX21 have been linked to T cell immune and innate immune (Dong et al., 2016; Saito et al., 2012); FHL2 was involved in diet-induced obesity (Clemente-Olivo et al., 2021); GPNMB was associated with lipogenesis in white adipose tissue and obesity and insulin resistance (Gong et al., 2019), which could reflect the difference in neuronal development, growth, lipid and cholesterol metabolism and immunity between the BMX pig and the CWB.


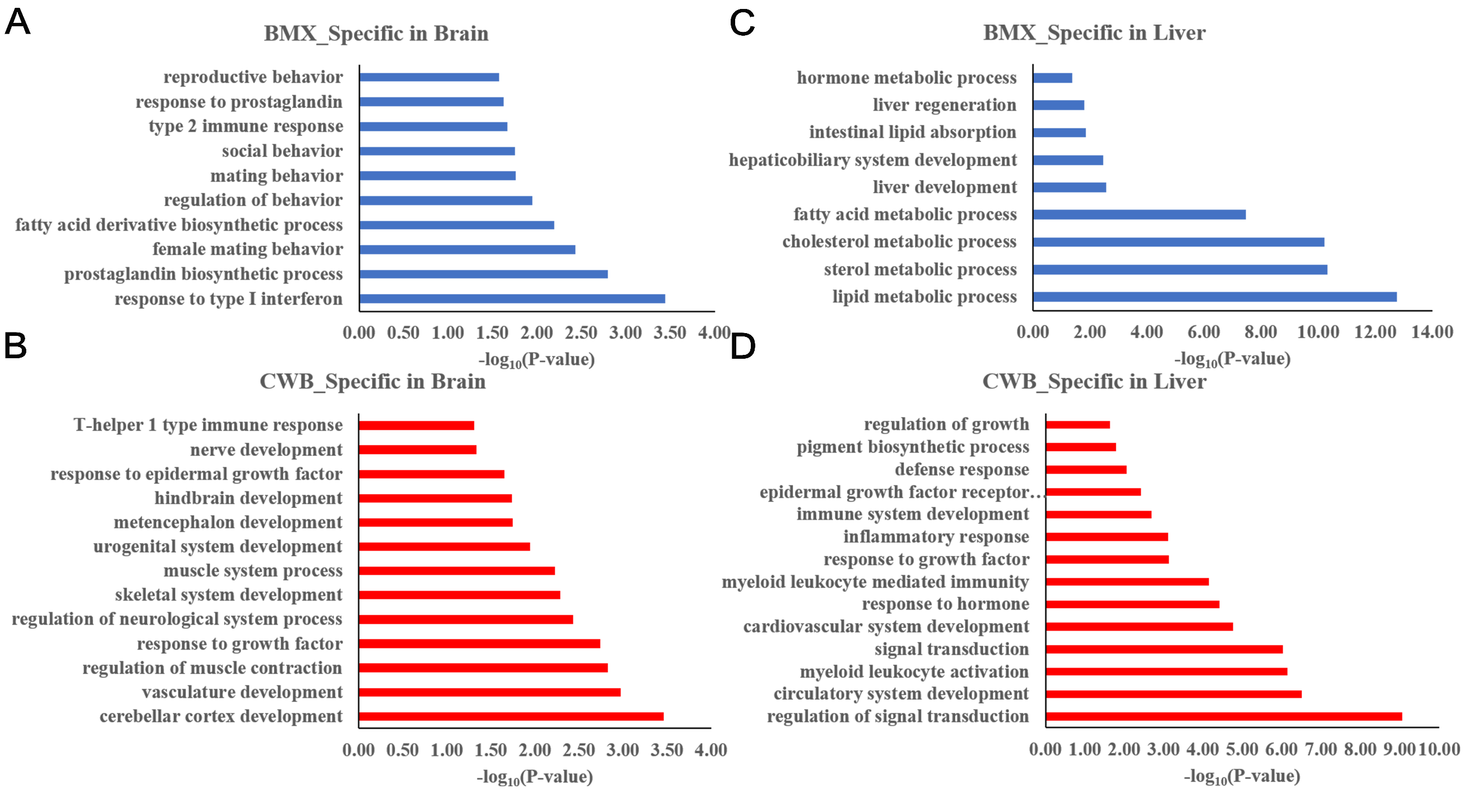


Figure S3. Enriched Gene Ontology terms for differentially expressed genes between BMXs and CWBs in the brain and liver. (A) BMX specific in brain. (B) CWB specific in brain. (C) BMX specific in liver. (D) CWB specific in liver.


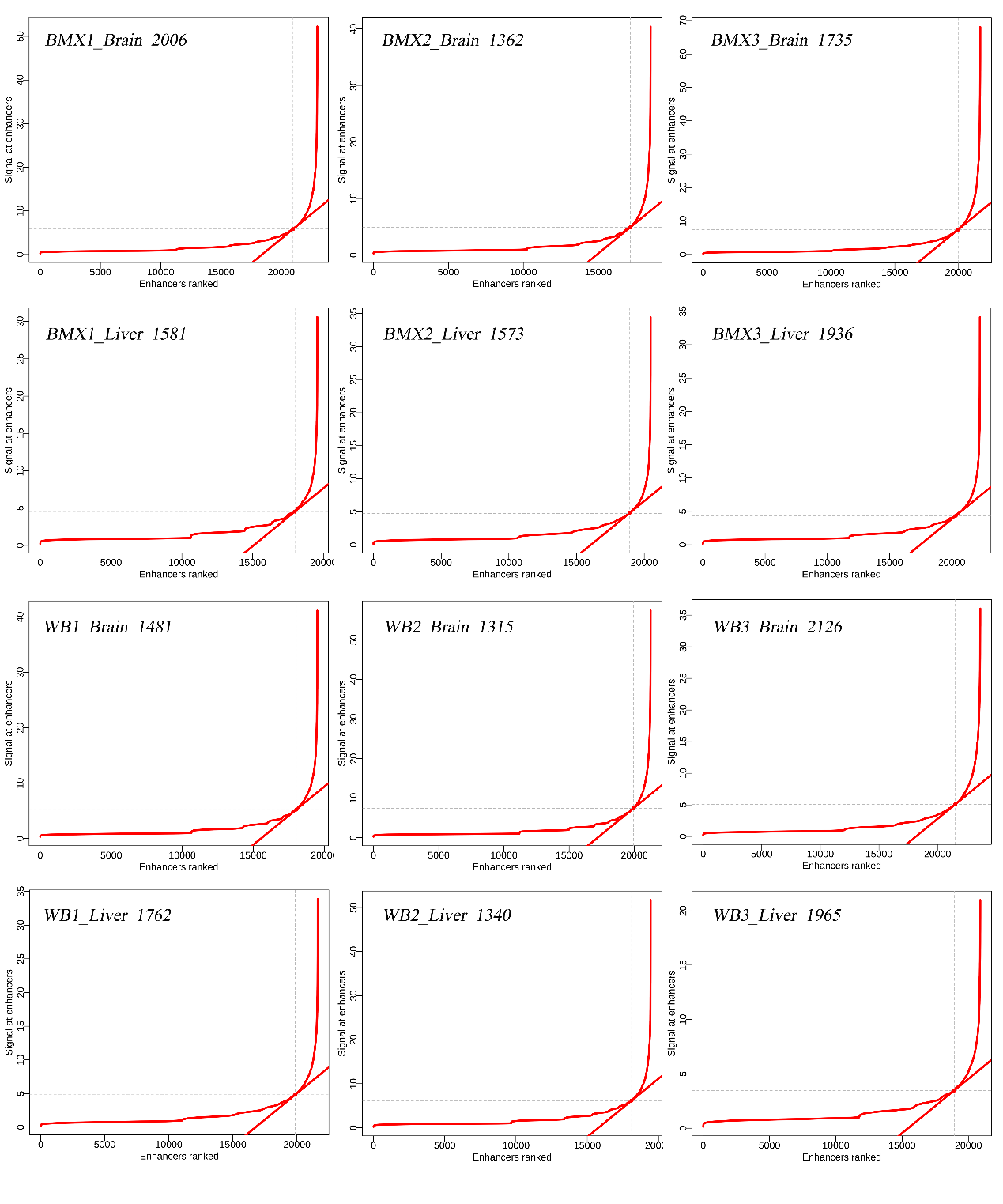


Figure S4. The identification of super enhancers in all H3K27ac-targeted ChIP-Seq samples


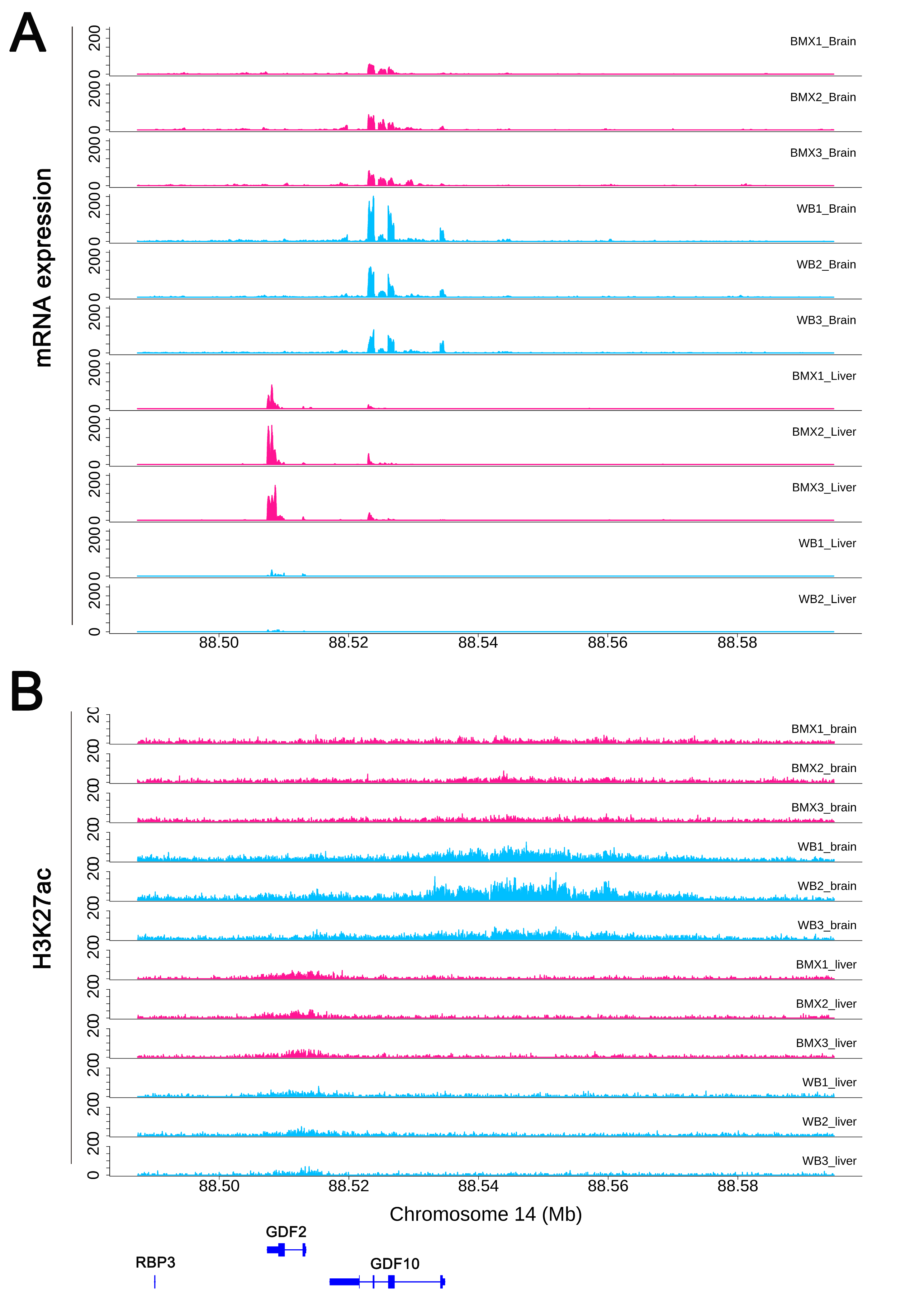


Figure S5. The tracks of H3K27ac activity and gene in the representative super enhancer that shows differential activity between BMXs and CWBs for reference (H3K27ac: chr14:88513242-88574880 Gene: *GDF10*).


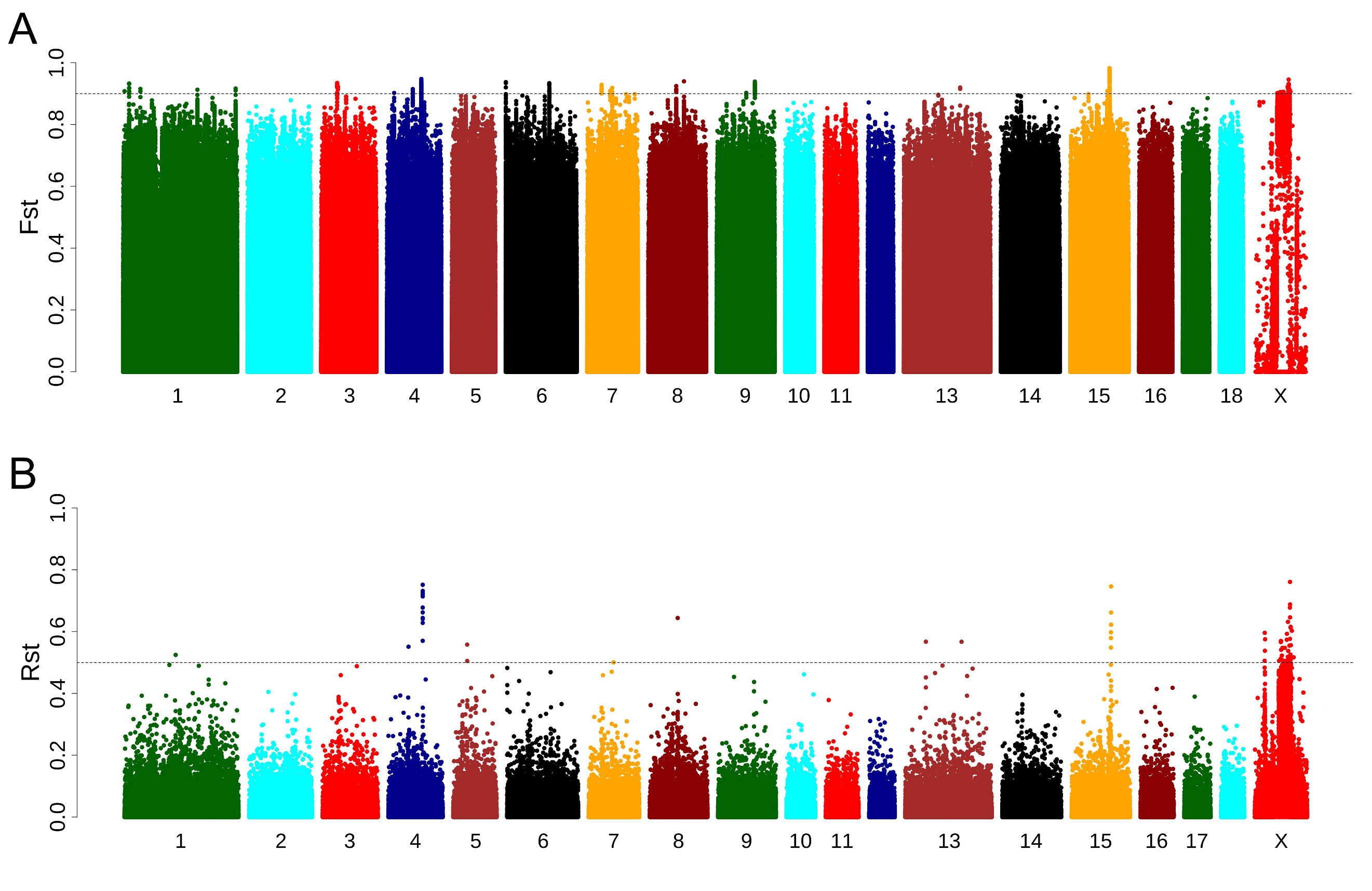
 Figure S6. Population differentiation of SNPs and STRs between domestic pig and wild boars. Manhattan plots showing the distributions of genome-wide Fst values (SNPs) and Rst values (STRs) between domestic pigs and wild boars.


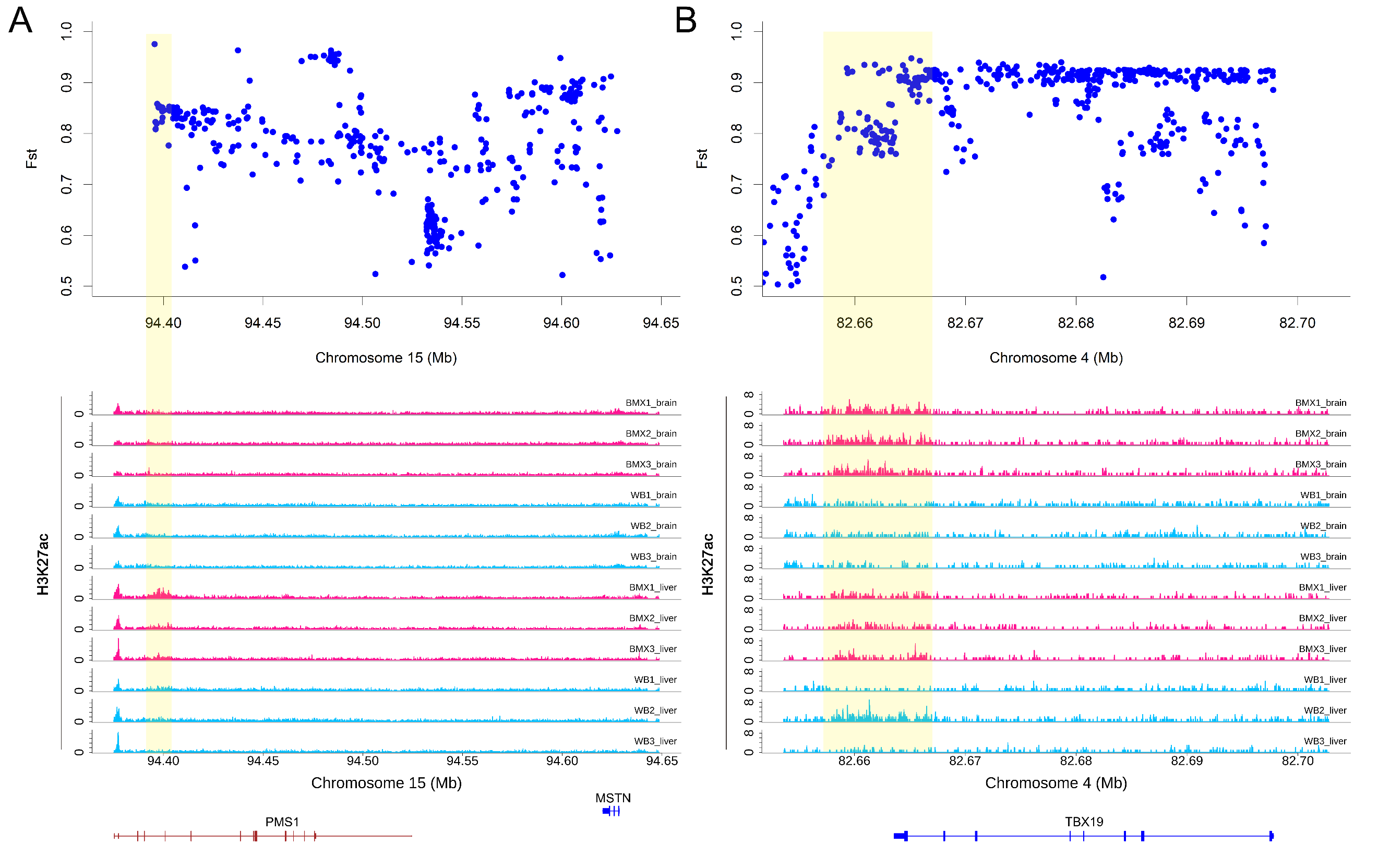


Figure S7. Overlap of differentially active H3K27ac peaks with previously reported differentiated loci and the candidate genes. (A) The BMX-high activity peak (chr15:94395185-94404081) was adjacent to MSTN. (B) There are three BMX-high activity peaks (chr4:82658612-82659843, chr4:82661072-82661538 and chr4-82664040-82664615) located near TBX19. The yellow shading marks the differential H3K27ac region between BMX and CWB.


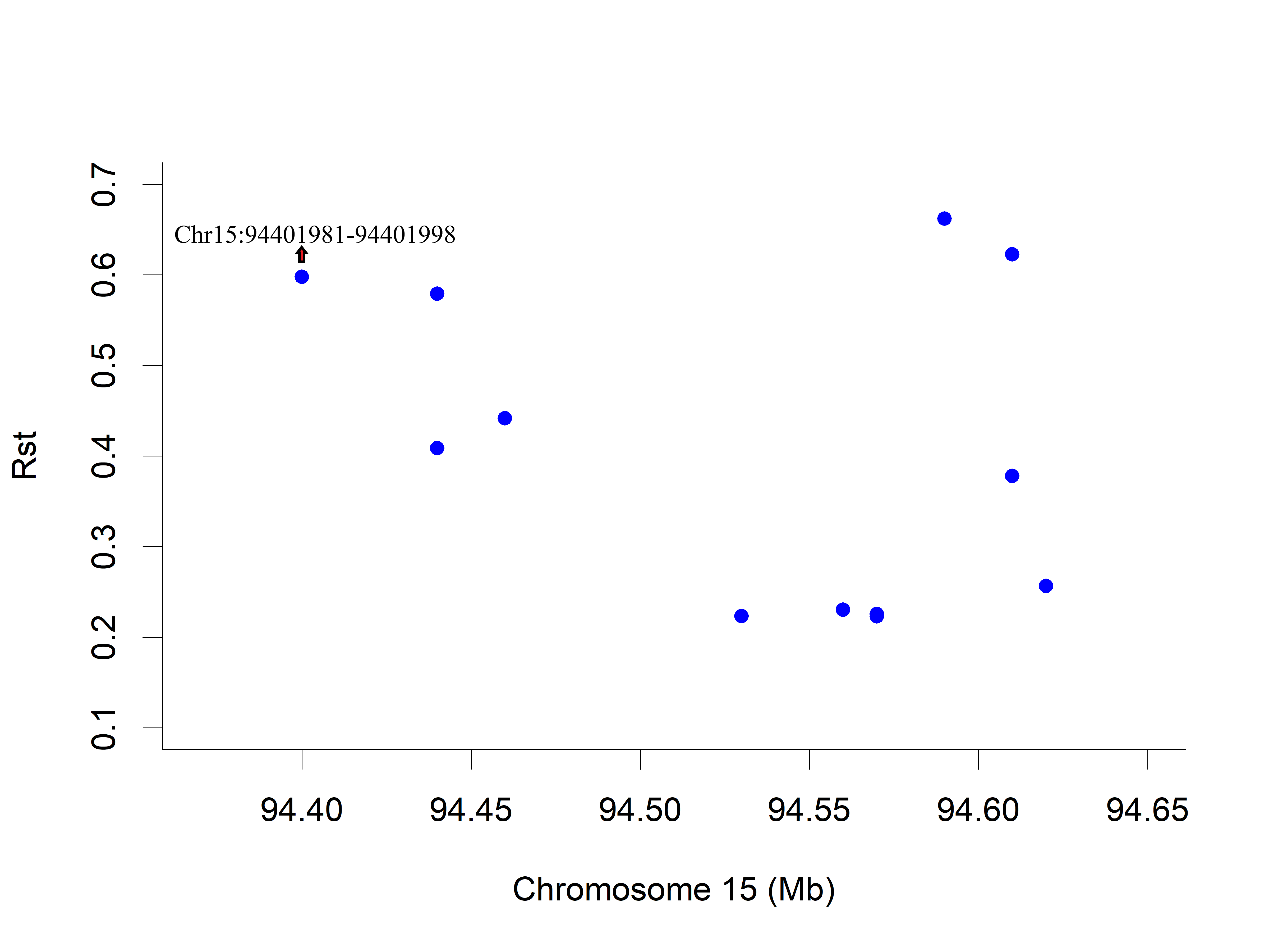


Figure S8. The fixation index (Rst) of STRs around *MSTN*. The position of the region represented by the abscissa is consistent with Figure S7A


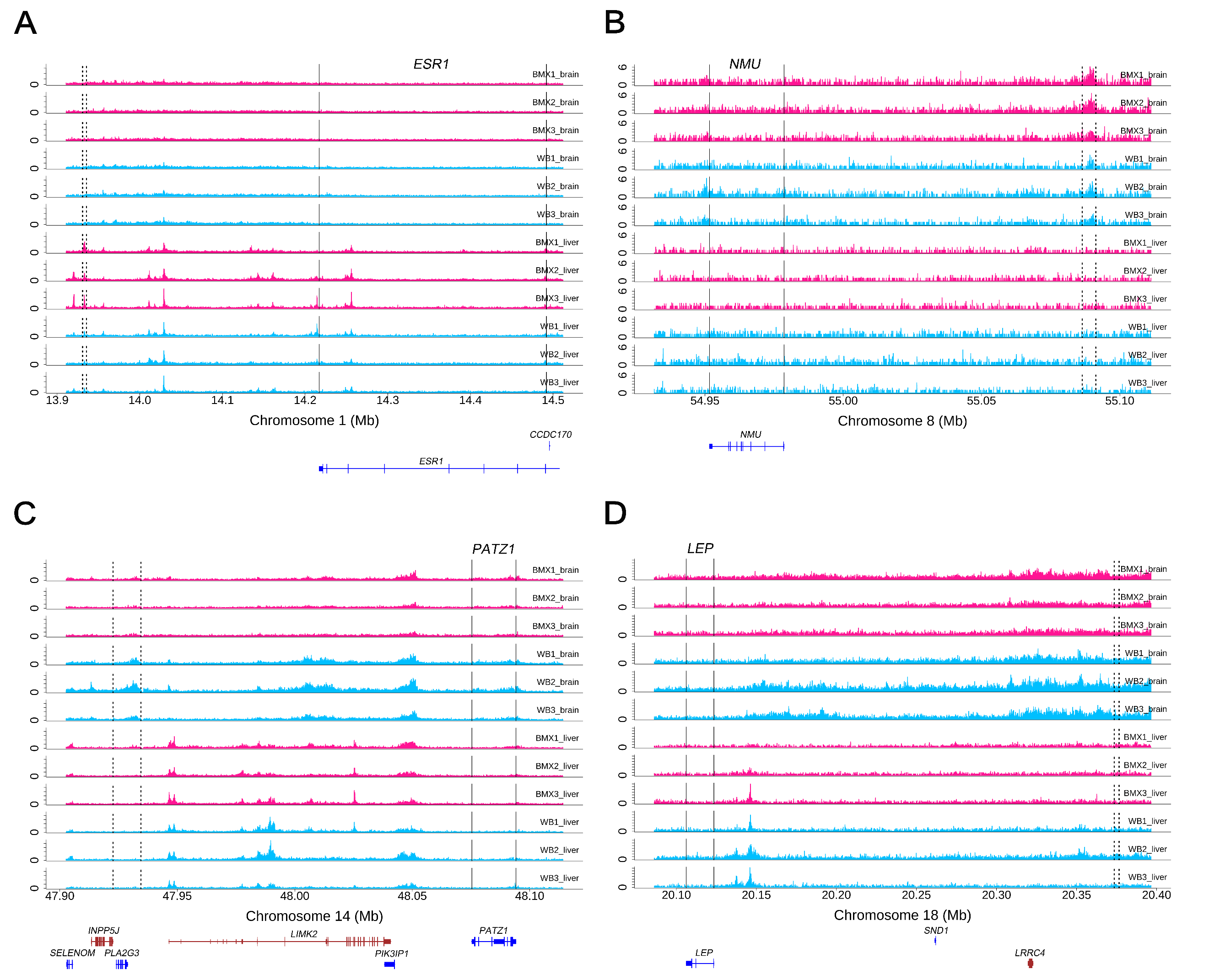


Figure S9. The tracks of H3K27ac activity in the four representative peaks (chr1:13930748- 13935472, chr8:55086422-55091330, chr14:47922638- 47934516 and chr18:20373534-20376561) that show differential activity between BMX and CWB for reference corresponding to *ESR1* (A), *NMU* (B), *PATZ1* (C) and *LEP* (D). The region between the dotted lines was the differential H3K27ac peak and solid lines was the gene.
